# Small molecule-based treatment approaches for intervertebral disc degeneration: Current options and future directions

**DOI:** 10.7150/thno.48987

**Published:** 2021-01-01

**Authors:** Amir Kamali, Reihane Ziadlou, Gernot Lang, Judith Pfannkuche, Shangbin Cui, Zhen Li, R. Geoff Richards, Mauro Alini, Sibylle Grad

**Affiliations:** 1 AO Research Institute Davos, Davos, Switzerland.; 2 Department of Orthopaedic and Trauma Surgery, University Medical Center Freiburg, Albert-Ludwigs University of Freiburg, Freiburg, Germany.; 3 Department of Biomedical Engineering, Medical Faculty of the University of Basel, Basel, CH.; 4 The first affiliated hospital of Sun Yat-sen University, Guangzhou, China.

**Keywords:** small molecules, discogenic pain, intervertebral disc, degeneration, inflammation

## Abstract

Low back pain (LBP) is a major reason for disability, and symptomatic intervertebral disc (IVD) degeneration (IDD) contributes to roughly 40% of all LBP cases. Current treatment modalities for IDD include conservative and surgical strategies. Unfortunately, there is a significant number of patients in which conventional therapies fail with the result that these patients remain suffering from chronic pain and disability. Furthermore, none of the current therapies successfully address the underlying biological problem - the symptomatic degenerated disc. Both spinal fusion as well as total disc replacement devices reduce spinal motion and are associated with adjacent segment disease. Thus, there is an unmet need for novel and stage-adjusted therapies to combat IDD. Several new treatment options aiming to regenerate the IVD are currently under investigation. The most common approaches include tissue engineering, growth factor therapy, gene therapy, and cell-based treatments according to the stage of degeneration. Recently, the regenerative activity of small molecules (low molecular weight organic compounds with less than 900 daltons) on IDD was demonstrated. However, small molecule-based therapy in IDD is still in its infancy due to limited knowledge about the mechanisms that control different cell signaling pathways of IVD homeostasis. Small molecules can act as anti-inflammatory, anti-apoptotic, anti-oxidative, and anabolic agents, which can prevent further degeneration of disc cells and enhance their regeneration. This review pursues to give a comprehensive overview of small molecules, focusing on low molecular weight organic compounds, and their potential utilization in patients with IDD based on recent *in vitro*, *in vivo,* and pre-clinical studies.

## Introduction

### Discogenic back pain

Globally, chronic low back pain (CLBP) symptoms occur in ~60-80% of people during their lifetime, which has a significant socioeconomic impact via reduced quality of life and work efficacy 1-4. CLBP is a multifactorial and complex clinical presentation, and symptomatic intervertebral disc (IVD) degeneration (IDD) is considered as the major cause of CLBP 5, 6. The IVD is a fibrocartilaginous tissue that lies between two vertebrae and functions as a shock-absorber. It includes the jelly-like nucleus pulposus (NP), the surrounding fibrocartilaginous annulus fibrosus (AF), and the cartilaginous endplate (CEP) anchoring the IVD to the corpus vertebrae. IVDs are crucial structural components that form a fibrocartilage joint allowing for slight intervertebral motion 7.

IDD features extracellular matrix (ECM) degradation, accelerated cartilaginous and bone remodeling, the release of proinflammatory cytokines, altered spine biomechanics, angiogenesis, and neoinnervation, altogether causing CLBP and disability 8-10. IDD can be induced or accelerated by mechanical stress, trauma, infection, genetic predisposition, or inflammation 10, 11. Due to the limited healing potential and harsh nutritional conditions of adult IVDs, IVD ECM degradation is irreversible and requires restoration if disc regeneration is pursued. Previous *in vitro* and* in vivo* animal and human studies showed cellular senescence as a critical mechanism in the progression of IVD aging, increased inflammation, elevated catabolism, and subsequently IDD 12-14. There is an unmet need for causative therapies especially for young patients affected by IDD that do not benefit from conservative treatments but, at the same time, do not qualify for spinal surgery. Therefore, the diagnosis and treatment of IDD in young patients would be a priority as in these disease stages the IVD still contains viable cells 15.

Most therapeutic options for IDD like analgesics, anti-inflammatory medications, and physical therapy are currently limited to symptomatic treatments, which only delay or mask the degradation process of the IVD. Surgical intervention is used as a last resort, with procedures such as total disc replacement or spinal fusion, which are associated with a substantial risk of intraoperative and postoperative complications 16. Recently, new strategies like stem cell, gene, and molecular therapy have been used for the regeneration of the IVD. Even though these methods opened new possibilities, they also have their limitations 17, 18.

Therefore, there is a strong demand to find new therapeutic agents (or utilize well-known drugs which were proven effective in treating other diseases) aiming to relieve discogenic pain and regenerate damaged IVDs through restoration of tissue homeostasis. In this regard, several small molecules have shown promising results as alternative therapeutic agents in *in vitro*, *in vivo*, and clinical studies 19, 20. These therapeutic agents demonstrate various phenomena to induce regeneration and prevent degeneration of the IVD, which include anti-oxidative, anti-inflammatory, anti-senescence, anti-apoptotic, anti-catabolic, and anabolic effects. This review is focused on low molecular weight organic compounds that have been investigated for their regenerative effects on IVDs. Furthermore, we will discuss how these small molecules may facilitate new treatment approaches for IVD regeneration.

### Anatomy of the intervertebral disc

**Figure 1:**
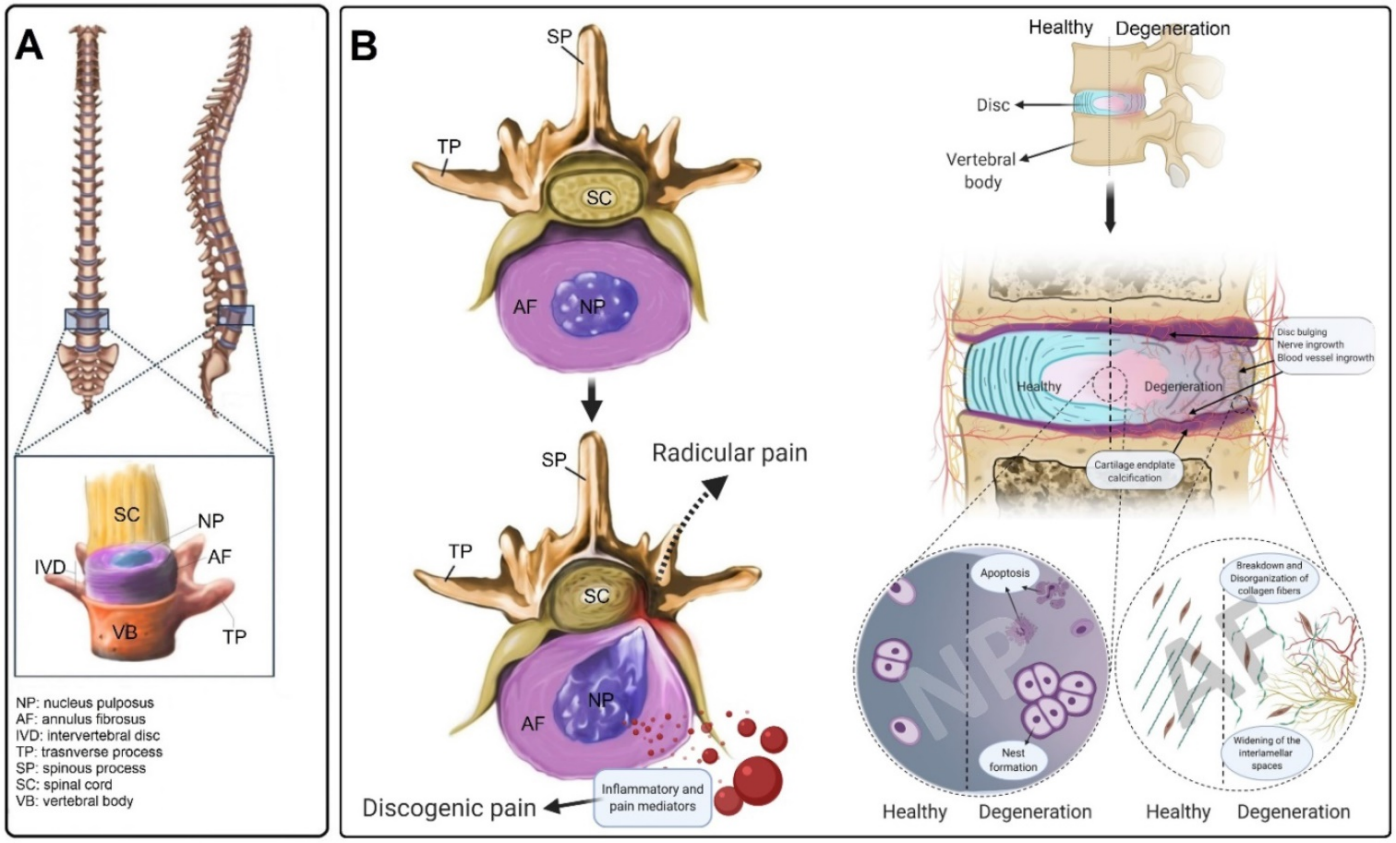
** The intervertebral disc structure and the hallmarks of IVD degeneration (IDD). (A)** The structure of the IVD and its anatomical location in the vertebral column. **(B)** Compared to healthy IVD, inflammation, blood vessel and neuronal ingrowth escalated in the degenerated disc. Moreover, the disorganization of collagen fibers and increasing of the interlamellar distances between collagen bundles in the annulus fibrosus were usually observed during the degeneration process that often results in disc bulging. During IDD, the number of apoptotic disc cells dramatically increased as well as the cell-cluster formation of nucleus pulposus (NP) cells. The calcification of the cartilage endplate and osteophyte formation occur in advanced degeneration. IVD degeneration is diagnosed when the degenerated IVD in the spine becomes symptomatic and causes discogenic pain. Additionally, the degenerated IVD (protrusion, bulging etc.) presses on spinal nerves, often producing radicular pain.

The IVD is the largest avascular structure in the human body that contains three main components; the soft mucoid NP core, the lamellar AF tissue that encloses the NP, and the CEPs which cover both top and bottom of the IVD (**Figure 1A**) 21. Different cell populations produce a unique composition of ECM, forming a special microenvironment for the IVD, which plays an important role in its functionality and mechanical properties 22. The high density of negatively charged proteoglycan (PG) molecules provides a capacity to absorb approximately three times their weight in water, giving the NP its mechanical resilience during compressive loading 23. The NP is circumferentially surrounded by the AF, a fibrocartilaginous tissue consisting of highly organized collagen fibers that are arranged in concentric layers 24. The AF is predominantly composed of both type I and II collagen and small quantities of PGs 25. The outer layer of the AF is mainly made of collagen type I (95%); however, the amount of this collagen type is significantly decreasing in an almost a linear negative gradient when approaching the NP, where it constitutes less than 5% of collagen type I 26. In contrast, an opposing pattern exists for collagen type II, decreasing in content towards the outer layers of the AF 26. The endplate is an osteochondral structure that consists of two parts, including CEP and bony endplate (BEP) that physically limit the NP and AF to their anatomical partitions. Along with its mechanically supporting role, the CEP controls the fluid exchange, as well as an exchange of nutrients or metabolic waste, and acts as a semipermeable barrier between discs and vertebrae 27.

### Damage, inflammation and IDD

The etiology of IDD is multifactorial and usually associated with genetic and environmental factors. 28. IDD often occurs when the balance between catabolism and anabolism of the ECM is disturbed by decreased ECM production and enhanced ECM degradation 29. The IVD (NP and AF) cells are responsible for keeping the balance between anabolic and catabolic processes including the synthesis, breakdown, and accumulation of ECM components 30. The quality of ECM composition and the IVD mechanical properties are determined by these cellular processes that are high energy demanding and require glucose and oxygen consumption. During the IDD process, the expression of inflammatory cytokines (i.e., IL-1 and TNF) in disc cells is increased, which subsequently up-regulates matrix remodelling. Through inflammatory matrix remodelling processes, the concentration of PGs and collagen type II is dramatically decreased, which is mainly mediated by two extracellular enzyme types: matrix metalloproteinases (MMP) and a disintegrin and metalloproteinase with thrombospondin motifs proteins (ADAMTS). Simultaneously, the amount of collagen type I is increased which altogether can change the ECM shear stresses 30. Moreover, an *in vitro* study showed that aggrecan, the major PG of the IVD, can inhibit neural ingrowth, which is associated with the development of CLBP 30, 31. Therefore, it is suggested that detrimental changes in the ECM are linked with discogenic pain.

Damage to the CEP can be another reason for IVD degeneration through both mechanical and nutritional factors. Damage to the CEP changes mechanical loading of the NP, stimulating metabolic disturbances in the disc 32. With increasing age, calcification of the endplate occurs, which may disturb its permeability and transportation of nutrients and other metabolites, leading to hypoxia and an acidic pH. This impairs the normal activity of IVD cells in synthesizing and supporting the ECM 33.

Inflammation is another factor that is thought to play an important role in the development of IDD 34. It is not known whether inflammation is the cause or consequence of disc degeneration and herniation. However, pro-inflammatory cytokines and chemokines, which are produced during both systemic and local inflammation, have been associated with IDD and lower back pain. Overproduction of chemokines and cytokines including interferon-gamma (IFN-γ), tumor necrosis factor-alpha (TNF-α), and interleukins (IL-1, 2, 4, 6, 8, and 17) by inflammatory cells present in the IVD can trigger the cascade of tissue degeneration. Moreover, several angiogenic and neurogenic factors (i.e., vascular endothelial growth factor, nerve growth factor) are also released during the IDD process, leading to blood vessel and nerve in-growth 35. It is hypothesized that endogenous factors, such as ECM breakdown products, can induce IVD inflammatory responses 36. Fibronectin, collagen, elastin, laminins, and low molecular weight hyaluronan are produced in response to an imbalance of homeostasis in ECM proteins. These products, in turn, induce an inflammatory response in the IVD 37-40. Finally, all these processes can lead to discogenic and/or radicular pain (**Figure 1B**).

## Small molecules and IVD regeneration

In the context of this review, we focus on small molecules as low molecular weight (<900 daltons) compounds, including synthetic or natural products 41. In the area of pharmaceuticals, small molecules are defined as compounds that bind to certain biological macromolecules and help regulating a particular biological process. The upper molecular weight limit for a small molecule, which requires rapid diffusion across the cell membrane and digestive system absorption, is 900 daltons. Basically, the molecules larger than 550 daltons face more challenges for absorption, while there are some up to 900 daltons that successfully cross barriers 42, 43. Historically, they were provided as drugs (such as celecoxib) to modulate different cell processes. In the last few years, several small molecules that can selectively regulate cell fate and signaling pathways have been developed 44. Indeed, small molecules have several advantages and only few limitations compared to large molecular compounds, as outlined in Table S1. Here we focus on recent advances in the use of small molecules that are effective in the regeneration of IVD cells through attenuation of inflammation, cell damage, and stimulation of anabolic processes. These molecules are listed in Table S2, and their mechanism of natural action is summarized in **Table 1.** In addition, we will discuss new strategies and approaches which were used in recent studies and the future direction for using these molecules to regenerate IVD cells. *In vitro, in vivo,* and clinical studies related to the discussed small molecules are listed in **Table 2.** The effective *in vitro* concentrations of different small molecules for regeneration of disc cells are listed in Table S3.

### Anti-inflammatory effects of small molecules

Although detailed pathways and molecular interactions between discogenic pain, disc degeneration, and inflammation remain to be elucidated, some inflammatory cytokines and related pathways are known as potential targets for therapies in IDD 19. Pro-inflammatory cytokines such as IL-1 and TNF-α are key cytokines, triggering ECM degeneration through activation of NF-κB and p38/MAPK pathways. One of the most important cell signaling pathways that seem to play a crucial role in IDD is MAPK signaling. Through this pathway, both matrix synthesis and degradation are modulated in the IVD by influencing PG degradation as well as by changing anabolic and catabolic gene expression levels 45. Particularly, the PG metabolism is regulated by p38/MAPK/extracellular signal-regulated kinase (ERK) signaling pathways, as treatment with inhibitors of p38 or ERK considerably attenuated the cytokine-induced decrease in synthesis and release of PG. Moreover, ERK can activate the Wnt/β-catenin signaling pathway, which may contribute to the pathogenesis of IDD. Activation of p38 and ERK, which was shown to be higher in degenerated IVD cells can enhance apoptosis induced by experimental loading stress 45. During IDD development, several growth promoting factors such as insulin-like growth factor 1 (IGF-I), platelet-derived growth factor (PDGF), and fibroblast growth factor (FGF) exert their beneficial effects (i.e., mitogenic action) via activating ERK by phosphorylation and subsequent DNA synthesis in degenerated disc cells, indicating that MAPKs may be involved in metabolic processes in the IVD 46. TNF-α is a cytokine that has been closely related to IDD. This cytokine has two receptors (TNFR1 and TNFR2) that bind the ligand with high affinity. Upon TNF binding to TNFR1, two distinct signaling complexes can be activated, 1) the anti-apoptotic complex I, and 2) the death inducing signaling complex (complex II). Signaling downstream of anti-apoptotic complex I is mediated by NFκB/MAPK signaling pathways. IL-1 is another cytokine that has strongly been linked to IDD. Among 11 cytokines of the IL-1 family, IL-1α and IL-1β are the most studied cytokines regarding IDD. Like TNF, IL-1α and IL-1β can activate NFκB and MAPK signaling pathways 47. As downstream effects, these cytokines activate MMPs and ADAMTS, which finally elevate ECM degradation.

**Figure 2:**
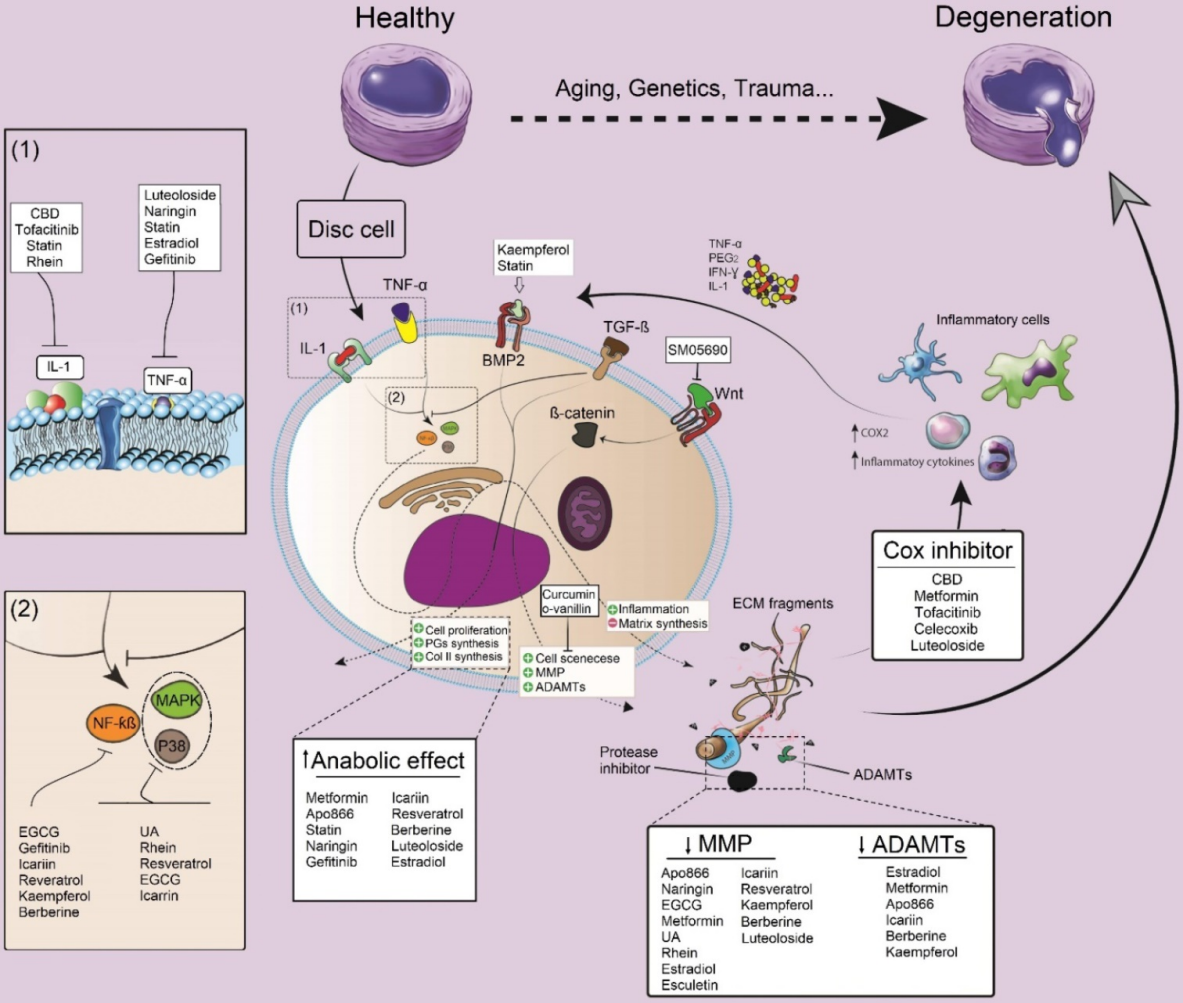
** The modulatory effect of small molecules on inflammation, anabolic and catabolic processes and their impacts on IDD.** Pro-inflammatory cytokines including IL-1 and TNF-a, are the key cytokines triggering IVD matrix degeneration through activation of NF-κB and P38/MAPK pathways. Moreover, these cytokines activate MMPs and a disintegrin and metalloproteinase with thrombospondin motifs (ADAMTs) which finally will elevate ECM degradation. Small molecules can prevent IDD progression by inhibiting the activity of pro-inflammatory cytokines and the subsequent factors (NF-κB and P38/MAPK), or modulating the anabolic, catabolic and even some alternative pathways such as BMP-2 or Wnt pathways. IL: interleukin; ADAMTS: a disintegrin and metalloproteinase with thrombospondin motifs; MMPs: matrix metalloproteinase; CBD: cannabidiol; ECGC: Epigallocatechin gallate; NF-kB: nuclear factor kappa-light-chain-enhancer of activated B cells; MAPK: MAP Kinase; UA: urolithin A, TGF-β: transforming growth factor-beta; Cox: cyclooxygenase; PGE2: prostaglandin E2; IFN-γ: interferon-gamma; TNF-α: tumor necrosis factor-alpha.

In various *in vitro* studies, small molecules, including naringin, cannabidiol (CBD), epigallocatechin gallate (EGCG), curcumin, icariin, resveratrol, berberine, and tofacitinib showed an impact on the downregulation of IL-1 and TNF-α levels in IVD cells (**Figure 2**). According to previous literature, icariin, resveratrol, and EGCG can inhibit NF-kB and p38/MAPK signaling pathways, thereby modulating inflammatory responses and preventing the development of a degenerative cascade 48-51. Gefitinib, kaempferol, and berberine are other small molecules that exclusively block the NF-kB signaling pathway 20, 52, 53. On the other hand, intracellular p38/MAPK signals could be blocked by rhein and urolithin A *in vitro* 54, 55.

Lang *et al.* investigated the effects of tofacitinib in an inflammatory and degenerative bovine IVD organ culture model. Tofacitinib citrate (2.5 mg/mL) was added daily to the culture medium to simulate a systemic application of the drug. The results showed that tofacitinib could slow down the degenerative response and reduce inflammation in the organ culture model by selectively inhibiting the Janus kinase 3 (JAK3) pathway 56. A recent *in vitro* study showed that luteoloside, a flavonoid glycoside, could suppress inflammatory factors, such as TNF-α and IL-6, in IL-1β-primed NP cells through inhibition of the NF-κB signaling cascade. They demonstrated that luteoloside promoted the nuclear factor erythroid 2-related factor 2 (Nrf2) translocation to the nuclei; Nrf2 can act through activation of the Nrf2/HO-1 (heme oxygenase-1) signaling in NP cells and mitigate inflammation by suppressing the NF-κB signaling cascade and by anti-apoptotic function 57.

Cyclooxygenase-2 (COX-2), which regulates prostaglandin E2 (PGE2) synthesis, is another candidate for modulation of inflammatory responses in IVDs. Chen *et al.* showed that pre-treatment with CBD suppressed the production of COX‑2 and inflammatory cytokines (i.e. IL-6 and IL‑1β) in NP cells 58. Moreover, metformin, luteoloside, and icariin exhibited their anti-inflammatory impact on IL-1 primed NP cells via inhibition of COX‑2 and inducible nitric oxide synthase (iNOS) expression, leading to a decreased synthesis of PGE2 51, 57, 59. Suzuki *et al.* used the JAK antagonist tofacitinib for pre-treatment of rat AF cells and then cells were incubated with inflammatory cytokines such as IL-6. Tofacitinib significantly decreased the expression level of COX-2 and, subsequently, the production of PGE2 60. Celecoxib is one of the most popular anti-inflammatory small molecules which has recently been used for regeneration of the IVD. Of the selective COX-2-inhibitors, celecoxib was the first on the market and has been in clinical use since 1999. In a preclinical canine study, local delivery and sustained release of this small molecule (as celecoxib-loaded microspheres) in the IVD showed promising results in the control of inflammation, attenuation of discogenic pain and inhibition of IDD development 61 (**Figure 2**).

### Anti-apoptotic effect of small molecules

**Figure 3:**
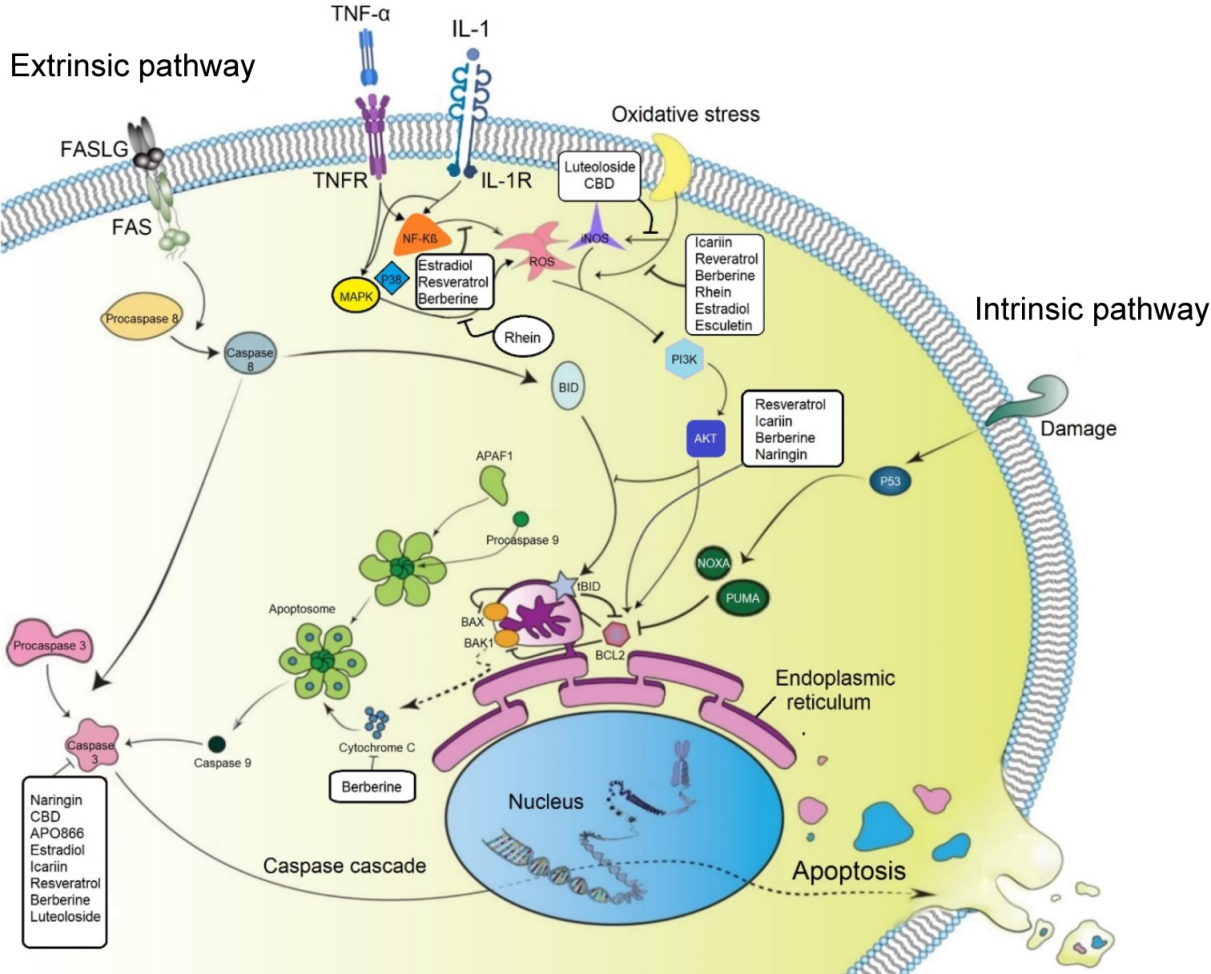
** The effect of small molecules on programmed cell death (apoptosis) and oxidative stress in degenerated IVD cells.** Both intrinsic and extrinsic pathways of apoptosis are playing critical roles in IVD cell degeneration. Small molecules can increase the expression level of Bcl-2, leading to inhibition of the intrinsic pathway by its inhibitory effect on BAX and BAK1. BAX and BAK1 control the release of cytochrome C from mitochondria to cytosol or bind to Apaf-1, leading to inhibition of caspase 9 activity. Several small molecules can also decrease the expression level of caspase 3 in disc cells and subsequently inhibit apoptosis. Inflammatory cytokines and oxidative stresses can also increase the number of apoptotic cells in degenerated IVD cells by activating the ROS-mediated PI3K/Akt pathway. Production of ROS and the expression level of related products such as iNOS can be suppressed by different small molecules. TNF-α: tumor necrosis factor-alpha; IL: interleukin; NF-kB: nuclear factor kappa-light-chain-enhancer of activated B cells; MAPK: MAP Kinase; Bcl2: B-cell lymphoma 2; Bax: BCL-Associated X; BAK1: BRI1-associated receptor kinase 1; PI3K: phosphatidylinositol 3-kinase; Akt: Protein Kinase B; ROS: reactive oxygen species; iNOS: inducible nitric oxide synthase; CBD: cannabidiol.

Therapeutic regulation of transduction pathways can modulate the programmed cell death (PCD) process, which is considered to play a significant role in IVD cell degeneration 62. Several studies investigated the anti-apoptotic effect of small molecules in IVD cells (**Figure 3**). Recent studies showed that naringin and icariin could upregulate the cellular concentrations of anti-apoptotic protein B-cell lymphoma 2 (Bcl-2) and downregulate the apoptotic effect of promoter proteins, including cleaved caspase 3 and BCL-associated X (Bax) to modulate the apoptotic rate of human NP derived cells 63, 64. Furthermore, after 4 hours of treatment with icariin, the phosphatidylinositol 3-kinase/protein kinase B (PI3K/AKT) signaling pathway was significantly activated. Based on previous studies, activation of this signaling pathway is correlated to anti-apoptosis and anti-oxidative stress; therefore, the anti-apoptotic effect of icariin is linked to the PI3K/AKT signaling pathway 63, 64. Chen *et al.* confirmed the protective effect of CBD on hydrogen peroxide-induced apoptosis. They identified that CBD increased cell viability and reduced apoptosis in NP cells after exposure to hydrogen peroxide by reducing the expression level of caspase 3 and promoting the Bcl-2 protein expression 58.

The effect of 17 beta-estradiol (E2) was also assessed on isolated NP cells from healthy rats and their intact IVDs, which were cultured with or without TNF-α. It was shown that in NP cells, E2 significantly increased matrix macromolecules expression, telomerase activity, and cell proliferation potential but attenuated senescence markers (p53 and p16). P53 plays a critical role in intrinsic pathways of apoptosis; therefore, the reduction of this marker could lead to a decrease in apoptosis 65. Rhein acts through several closely interacting pathways affecting apoptosis. Rhein blocks the p38/MAPK pathway which in turn activates the PI3K/AKT parallel signaling pathways. Consequentially several downstream pathways are activated which regulate the cell cycle and apoptosis 66. Therefore, the therapeutic potential of rhein as a multitarget molecule is due to its synergistic modulation of multiple pathways 67.

Resveratrol is another small molecule which could induce anti-apoptotic genes (e.g., Bcl-2) and simultaneously reduce the expression level of pro-apoptotic genes such as Bax or caspase 3. Moreover, when LY294002 was used as a strong inhibitor of PI3K/AKT, the anti-apoptotic effects of resveratrol in IL-1β-primed NP cells were attenuated 68. Therefore, it can be concluded that resveratrol activated the PI3K/AKT signaling pathway, which in turn downregulated NP cell apoptosis. Furthermore, another study showed that resveratrol could activate sirtuin 1 (NAD (+)-dependent deacetylase), which reduces apoptosis in degenerated human NP cells 69; while treatment with LY294002 again increased the rate of apoptosis. This study also suggested that resveratrol could increase the survival rate of degenerative human NP cells by activation of sirtuin 1 through the PI3K/AKT anti-apoptotic signaling pathway 69.

The protective effect of berberine by inhibiting NF-κB activation has been shown on IL-1β stimulated human NP cells undergoing apoptosis 70. The modulatory effects of berberine on the expression levels of anti-apoptotic protein (Bcl-2), activation of caspase 3, pro-apoptotic Bax, Bak, and release of cytochrome c have also been reported 71.

Pyroptosis is another form of programmed cell death which can lead to production of proinflammatory mediators 72. This process is mediated by nod-like receptor protein 3 (NLRP3) inflammasome, and it has been shown that suppressing the activation of NLRP3 inflammasome could diminish the IDD process 73. New evidence indicates that microorganisms such as *Cutibacterium acnes* can induce inflammatory response (IL-1β) and initiate IDD. The number of NLRP-positive cells significantly increased in *C. acnes* infected disc tissue, which suggested that pyroptosis activation may be induced by *C. acnes* 73. A recent study showed that dexmedetomidine, a sedative small molecule drug, inactivated NLRP3 through the suppression of NF-κB and JNK signals, subsequently alleviating pyroptosis during inflammation and IDD 74.

### Anti-oxidative effect of small molecules

During IDD, usually excessive reactive oxygen species (ROS) are produced and released locally, suggesting a contribution of oxidative stress to the degeneration process and opening a new horizon regarding the pathogenesis of IDD. ROS, as active mediators, are involved in various cell signaling pathways and cell metabolisms, including matrix degradation, inflammation, apoptosis, autophagy, and senescence of IVD cells. Moreover, ROS can change the structure of matrix proteins in NP, AF, and CEP leading to the impairment of the IVD's mechanical function and acceleration of IVD degeneration processes 75. Therefore, a therapeutic option for regulation of oxidative stress in disc cells could be a novel strategy for IVD regeneration. Apoptosis can be triggered by oxidative stress during IDD which has been elaborated in several studies. IL-1β treated NP cells produce more ROS in comparison to untreated cells, which consequently decreases the PG levels and triggers apoptosis 76. Moreover, the ratio of apoptosis is increased in NP cells exposed to hydrogen peroxide and the expression levels of ECM proteins such as aggrecan and type II collagen are decreased 77. Notably, the detrimental effects of oxidative stress on the cells may be efficiently prevented using anti-oxidative agents, such as different small molecules that protect IVD cells from apoptosis (**Figure 3**). For instance, the effect of naringin on oxidative stress-induced apoptosis was investigated in rat NP-derived mesenchymal stem cells (MSCs). The findings showed that naringin had protective effects against hydrogen peroxide induced NP cell apoptosis. The potential mechanism of naringin to alleviate apoptosis may be due to the activation of the ROS-mediated PI3K/AKT pathway 63. In the same way, the protective effect of CBD on NP cells against oxidative stress was also reported. The results demonstrated that the pre-treatment with CBD suppressed the expression level of iNOS, which activated PI3K/AKT signaling pathway 58. Another study by Krupkova *et al.* evaluated the anti-oxidative effect of EGCG on human IVD cells exposed to hydrogen peroxide. Their results demonstrated that survival of the treated disc cells by EGCG under severe oxidative stress was considerably enhanced in comparison to the control cells, which happened through activation of PI3K/AKT pathway and inhibition of cytochrome c release from mitochondria 78.

The inhibitory effect of E2 on ROS generation was studied by several investigators. ROS/NF-κB pathway of rat NP cells is affected by the interaction of estrogen receptor and E2, which inhibits TNF-α-induced premature senescence 65.

Cryopreservation can be used to allow the storage of cells over prolonged periods of time. While cryopreservation at -196°C would render IVD cells metabolically inactive, cells usually suffer insults during freeze-thawing such as the generation of ROS 79. For this reason, the effect of icariin (25 µM) as an addition to cryopreservation media was investigated by Chan *et al.* They found that icariin improved the viability and function of human NP derived stem cells by preserving the phenotype after thawing the cells 80. The increased activity of glutathione peroxidase (GPx) and superoxide dismutase (SOD) can explain the oxidation resistance, which provides oxidative stress protection to the cryopreserved cells. Several studies investigated the anti-oxidative effect of resveratrol on human, rat, and bovine NP cells *in vitro*. The protective effect of resveratrol is due to the stimulation of sirtuin 1 and the PI3K/AKT pathway which was activated in different settings 81-85. Also, icariin could inhibit induced oxidative stress in NP cells *in vitro* 64. Luo* et al*. observed that the production of ROS under hydrogen peroxide exposure was down-regulated with berberine, which protected human NP cells against oxidative stress-induced apoptosis 86. A recent *in vitro* study showed that luteoloside could successfully suppress iNOS and modulate ROS production in IL-1β treated NP cells 57.

Although most of the studies showed an anti-oxidative effect of these small molecules on IVD cells, there is not enough *in vivo* and preclinical evidence to support the efficiency of these molecules to retard the process of IDD. Further *in vivo* and clinical studies are required to develop effective anti-oxidative therapies for IVD regeneration.

### Anabolic and anti-catabolic effect of small molecules

Several studies assessed the anabolic and anti-catabolic effect of small molecules on IVD cells and reported their beneficial impacts on IDD (**Figure 2**). Li *et al.* evaluated the influence of naringin on the growth of degenerative NP cells and its regenerative effects on protein and gene expression. Naringin treatment elevated the protein expression of collagen type II, aggrecan, and SOX6, and decreased the gene expression of MMP3 87. Another study showed that naringin could promote the expression of anabolic genes such as collagen II, aggrecan, and reduce catabolic gene expression such as MMP13 to sustain the ECM 63.

Through an *in vitro* study, the TNF-α-induced production of MMPs and degradation of collagen II have been investigated, and the anti-senescence and anti-catabolic effects of urolithin A have been confirmed 88. Estradiol can increase the anabolic activity of NP cells and induce the downregulation of MMPs, indicating protective capabilities of estradiol, as shown *in vitro* 89. The down-regulation of the protein level of caspase-3, MMP3, and MMP13 and up-regulation of the protein level of type II collagen were closely related to the anti-degenerative mechanism 89. With the therapeutic application of E2 to degenerated CEP cells, increased expression of collagen II and aggrecan was noted. In addition, an increase in the TGF-β secretion was reported 90. Hua *et al*. demonstrated the anti-catabolic effect of icariin by decreased MMP and ADAMTS gene expression in human NP cells stimulated by IL-1β 51. An anabolic effect of 200 μM resveratrol and decrease of the catabolic effects of pro-inflammatory stimuli (IL-1β) added to bovine NP cells was also reported by Li *et al* 82. The anabolic effect of resveratrol in cell culture could be reproduced in an *in vivo* study by Kwon *et al*., who induced degeneration by annulotomy in rabbit discs followed by two intradiscal injections of resveratrol or carrier (DMSO) percutaneously. The regeneration of discs was assessed by magnetic resonance imaging (MRI), real-time polymerase chain reaction (RT-PCR), and histological analysis. An increased aggrecan and decreased MMP13 gene expression in the treatment group compared to the carrier was observed, and increased matrix PG production was confirmed by histology 91. The effect of statins on the homeostasis of disc cells was also investigated in previous studies. It has been shown that hydrophilic statins had more regenerative potential on NP cells than lipophilic statins. They also showed that hydrophilic statins increased the expression of type II collagen and SRY-box transcription factor 9 (SOX9) in a lower dosage than lipophilic statins.

It has been shown that the expression level of anabolic genes in NP cells increased by metformin, while the expression of catabolic genes considerably decreased 92. Gefitinib, a small molecule which inhibits epidermal growth factor receptor (EGFR), was also investigated for its potential effects on IDD regeneration. In an *in vitro* study, gefitinib at 10 µM was administered 30 minutes before the treatment of rat NP cells with 10 ng/ml TGF-α. After 48h of treatment with TGF-α, RNA and protein analyses were performed. It was concluded that gefitinib caused therapeutic inhibition of EGFR signaling thereby inhibiting IVD degeneration and enhancing IVD matrix synthesis in TGF-α treated NP cells 20. The modulating effect of tofacitinib on anabolic and catabolic processes was also investigated in rat and human degenerated IVD cells. Tofacitinib was used to pre-treat disc cells for 30 minutes, followed by incubation with soluble IL-6 receptor (Sil-6R) and IL-6. Tofacitinib decreased the expression of catabolic factors such as MMP13. The study showed the therapeutic potential of tofacitinib in lessening the development of IDD through suppressing the catabolic effects of IL-6 60. Two similar *in vitro* studies showed that luteoloside and APO 866 (daporinad) increased the content of ECM-related proteins such as type II collagen and aggrecan and reduced the expression level of MMP13 and ADAMTS5 in IL-1β primed disc cells 57, 93.

### Additional targets

#### Autophagy

Several investigations have demonstrated that autophagy occurs in IVD cells 94-96. Autophagy is a conserved cellular process that continues to occur in all types of cells throughout life. Through this well-coordinated and multi-step process, cells remove unnecessary or dysfunctional components. Usually, a low basal level of autophagy occurs in IVD cells, which was confirmed in cells isolated from non-degenerative adult rat discs 94. However, autophagy can considerably increase in degenerative IVD cells 97. This process can successfully reduce apoptosis in both NP and AF cells, leading to the attenuation of IVD degeneration 94. It has been demonstrated that nutrition deprivation markedly induced IVD cell apoptosis through the intrinsic pathway, whereas this process can be blocked by sirtuin 1 via acceleration of autophagy 98. Moreover, the suppression of autophagy by exposure of the cells to 3-methyladenine (an autophagy inhibitor) increased the apoptosis of the cells 99.

The impact of small molecules on autophagy was assessed in different *in vitro* studies. Shi *et al.* used autophagy markers (LC3 and Beclin-1) to assess the impact of APO866 on autophagy of NP cells as well as their apoptosis. The results showed that APO866 inhibited IL-1β-induced NP cell apoptosis by induction of autophagy. These findings showed the therapeutic potential of APO866 for IDD 93. Metformin activates the upstream regulator AMPK, which directly induces autophagy in NP cells in a dose- and time-dependent manner to block apoptosis 92. In a rat IDD model, the controlled-release of gefitinib protected IVDs from degeneration possibly through the modulation of the EGFR-autophagy axis which was shown to not only suppress cartilage matrix degradation but also boost type II collagen synthesis 20.

#### Mammalian target of rapamycin (mTOR)

In molecular signaling, the mammalian target of rapamycin (mTOR) acts as a negative regulator of autophagy. The mTOR is a serine/threonine kinase which regulates cellular activation such as cell growth and division, cell motility and cell survival. The mTOR exists in two distinct protein complexes including mTOR complex 1 (mTORC1) and mTOR complex 2 (mTORC2). Protein kinase B (known as AKT), an essential pro-survival mediator by suppressing apoptosis, regulates mTORC1 and mTORC2 100. It has been shown that the IVD cells would utilize the mTOR signaling and autophagy to cope with stressful conditions such as low oxygen, pH and nutrient concentration 101. Rapamycin is the primarily isolated mTORC1 inhibitor which can extend mammalian lifespan via inhibiting the cell cycle progression and lethal neoplastic diseases. Today, serious adverse effects of rapamycin including immunosuppression limited its extensive clinical use. A very recent study assessed the effects of mTOR inhibitors on human IVD cells. In this study, four different small molecules and mTORC inhibitors including INK-128, NVP-BEZ235, MK-2206 and curcumin were examined; the results showed the pharmacological modulation of mTOR signaling and autophagy increased the survival rate of IVD cells by suppression of apoptosis 102.

#### Anti-senescence effect

Senescent cells are non-dividing cells, which are still metabolically active. These cells have been shown to contribute to the catabolic shift in IVD tissue during degeneration by secreting the senescence-associated secretory phenotype (SASP) which contributes to a pro-inflammatory milieu 103. Curcumin and o-vanillin have been shown to both exert anti-inflammatory and anti-oxidative effects and act as senomorphic drugs. Cherif *et al.* observed a reduced number of senescent disc (NP and AF) cells and decreased SASP factors as well as an increase in cell proliferation after treatment with curcumin or o-vanillin. This effect was due to the selective induction of apoptosis in senescent cells without negative effects on the proliferating cells 103, 104.

#### Bone morphogenetic protein activators

Bone morphogenetic proteins (BMPs such as BMP-2 and BMP-7) have shown promise in IVD regeneration. Li *et al.* evaluated the effect of BMPs for regeneration of IVD both *in vitro* and *ex vivo*. Their results showed that BMP heterodimers could successfully upregulate the aggrecan and type II collagen gene expression, as well as glycosaminoglycan (GAG) synthesis of NP cells 93. The investigations of kaempferol and statins in the treatment of IDD have shown a potential regenerative effect via the BMP-2 signaling pathway 105, 106. Lovastatin (at concentrations ≥1 µM) could significantly enhance the expression of anabolic genes encoding BMP-2 and BMP-7 107. Although these small molecules show great potential in slowing down the progression of IVD degeneration via activation of the BMP-2 signaling pathway, more studies are needed to further elucidate their effects as BMP activators.

#### Wnt pathway Inhibitor

In IDD, increased Wnt signaling suppresses progenitor cell proliferation and induces apoptosis of NP cells 108. Moreover, it has been suggested that the Wnt signaling pathway is involved in fibrosis of the AF. SM04690, a small molecule inhibitor of the Wnt pathway, has been utilized in drug development for the treatment of IDD and osteoarthritis. SM04690 demonstrated regenerative properties in preclinical studies, including reduction of inflammation, inhibition of fibrosis, activation of NP cell proliferation, and production of ECM in a rat IVD model. These findings suggest SM04690 as a potential treatment for IDD 109.

### *In vivo* and clinical studies

**Figure 4:**
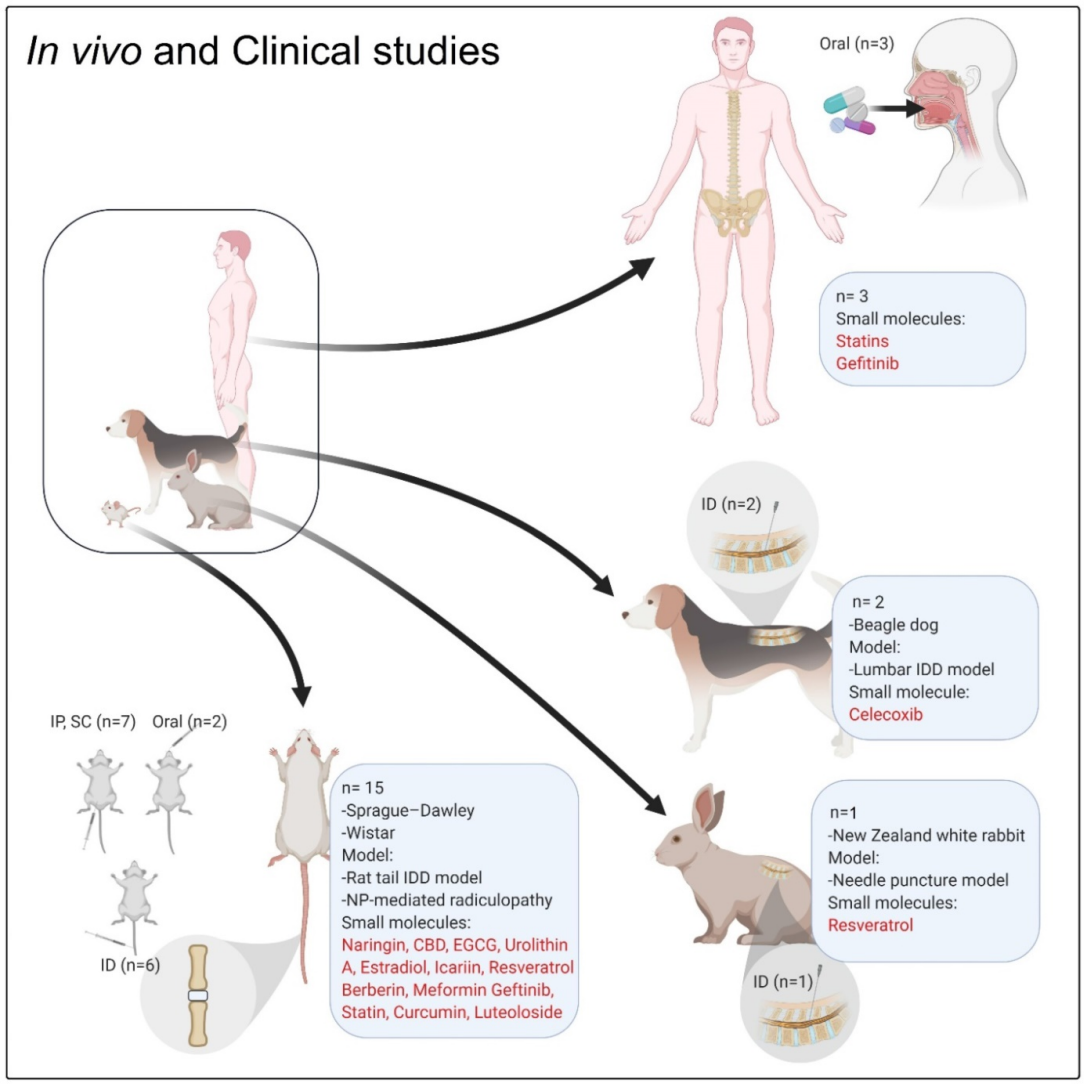
** The *in vivo* and clinical studies focused on the role of small molecules on IVD regeneration.** ''n'' denotes the number of *in vivo* or clinical studies, Abbreviation; IP: intraperitoneal injection; ID: intradiscal injection/delivery; CBD: cannabidiol; EGCG: epigallocatechin 3-gallate.

To date, several *in vivo* studies using small molecules and targeting IDD and related degenerative processes have been published (**Table 3, Figure 4**). All these *in vivo* studies showed promising results of different small molecules on IDD; however, there is still limited evidence for a regenerative impact of small molecules on degenerated disc tissue. Most of the cited studies showed the preventive effects of the drugs on IDD progression. Only one study showed that EGCG could reach the MRI and histological scoring levels measured in the sham group 50. Other studies showed that the symptoms or progression of IDD were alleviated in comparison with the vehicle-treated animals (negative control). Approximately 90% of *in vivo* investigations (**Table 3**) followed the animal cases up to 12 weeks, which is a very short-term follow-up and could explain such insignificant results 110.

**Figure 5:**
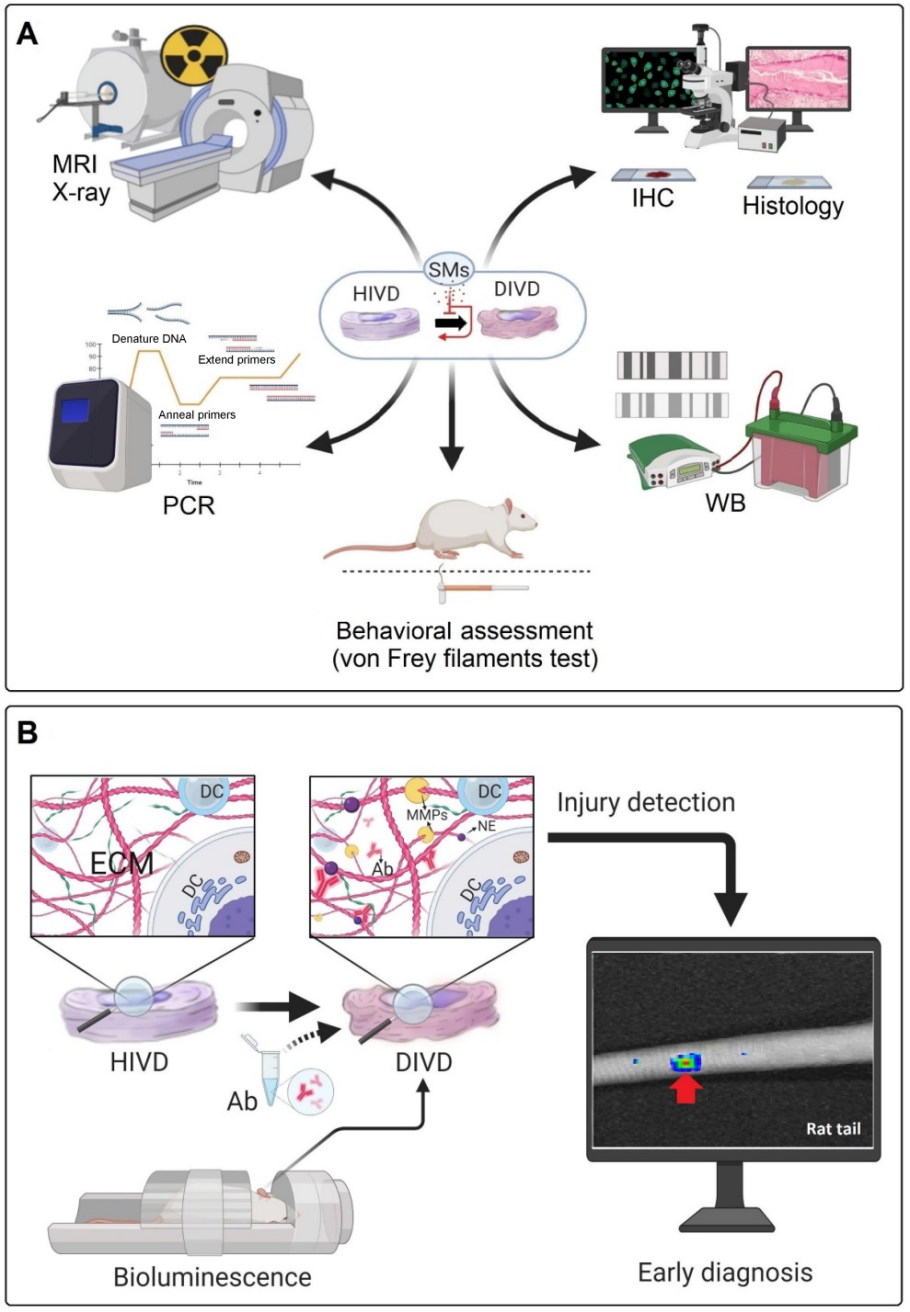
** Conventional diagnostic approaches and a proposed diagnostic method for detection of IDD. (A)** Frequently used diagnostic methods in several *in vivo* studies for the diagnosis of IVD degeneration and evaluation of treatment responses. **(B)** Specific antibodies (Ab) may be locally injected into disc areas through the proposed method to attach with the ECM neoepitopes (NE); which produced during early degeneration processes. Then, the whole body animal bioluminescence could be used to track the Abs-neoepitopes complex (rat tail IDD model). PCR: polymerase chain reaction; WB: western blot; MRI: magnetic resonance imaging; IHC: immunohistochemistry; HIVD: healthy intervertebral disc; DIVD: degenerated intervertebral disc; SMs: small molecules; Ab: antibody; NE: neo-epitope; ECM: extracellular matrix; DC: disc cell; MMPs: matrix metalloproteinases.

Most of the *in vivo* investigations used standard methods to analyze the regenerative process or the inhibition of IDD progression, including X-ray, MRI, histopathology and immunohistochemistry (IHC) (**Figure 5A**). However, these methods have some limitations to assess the beneficial effects of small molecules on IDD. For instance, MRI was used as a technique to analyze the disc height index (DHI) or the Pfirrmann grade in different treatment groups, whereby 3.0 Tesla (T) or lower (1.5T) clinical MRIs were frequently used (7 out of 10 *in vivo* studies on rodents). The 1.5T or 3.0T MRI and related image quality are not suitable for small animal imaging (mouse, rat, and rabbit), which may lead to an irreproducible scoring by the radiologists 111.

Small laboratory mammals are the main animal models for IDD 112, 113; according to **Table 3**, 16 out of 18 studies utilized rodent and lagomorph animal models (rat and rabbit) to explore the effect of small molecules on IDD regeneration or discogenic pain. However, significant limitations exist with using such animal models for IVD regeneration studies. For instance, in the rat tail models, the mechanism of disc injury is different due to different mechanical loading and persistence of notochordal cells which affect the regeneration processes. Furthermore, due to the smaller size of the disc, the diffusion of nutrition is different in comparison with the human disc. Another limitation is the lack of human-like longitudinal compression in the quadrupedal animal models 114. In previous studies, chondrodystrophic dogs (beagle) were used to analyse the effect of local delivery of celecoxib (CXB) on IDD 61, 115. Tellegen *et al.* studied the therapeutic effect and the safety of celecoxib-loaded microspheres administered in a canine IDD model. This study showed that there was no evidence of adverse effects on MRI or macroscopic evaluation of IVDs. The diagnostic analysis of NP PG content revealed that site-targeted and sustained administration of CXB inhibited IDD. Local delivery of the COX-2 inhibitor showed decreased neuronal growth factor and PGE2 tissue levels, which led to an inhibition of the inflammation and alleviation of pain 61. In another related study, the biocompatibility, safety and feasibility of poly(ε-caprolactone-co-lactide)-b-poly(ethylene glycol)-b-poly(ε-caprolactone-co-lactide) (PCLA-PEG-PCLA) hydrogel releasing celecoxib was investigated. The biocompatibility was evaluated by administering a subcutaneous injection in rats. The feasibility and safety were evaluated by administering an intradiscal injection to dogs suffering from early spontaneous IDD. Clinical improvement was achieved by reduction of back pain in 9/10 dogs, which was shown by clinical examination and owner questionnaires. The study demonstrated the effectiveness and safety of hydrogel-based celecoxib delivery 115. Chondrodystrophic dogs, as a large animal model, not only lose the notochordal cells following birth, but they also have a special phenotype (short and curved limbs) predisposing them to spontaneous disc degeneration. With respect to ethical concerns, the number of animals (N ranging from 12 up to 40, **Table 3**) is another limiting factor for the majority of *in vivo* studies, which can make it difficult to interpret the results due to high variations.

The behavioral assessment of discogenic pain (i.e., Von Frey filament test) in animal models is still in its infancy 110. Von Frey filament test provides a quantitative measurement of the paw withdrawal threshold 116. Krupkova *et al*. and Lin *et al.* used this method to analyze the effect of EGCG and resveratrol on IVD regeneration and related discogenic pain 50, 117. However, in other *in vivo* studies, it is unknown whether the regeneration of the IVD is correlated with the resolution of discogenic pain.

In a rat tail static compression model, Yurube *et al.* evaluated the presence of the MMP- and ADAMTS-cleaved aggrecan neoepitopes *in vivo,* using IHC. These neoepitopes produced by catabolic enzymes in the degeneration process could be used for early detection of IDD 118. As a proposed diagnostic approach, these antibodies could be modified and labeled with luminescence agents to be detectable via *in vivo* bioluminescence imaging. This idea might be developed in the future for early diagnosis of IDD and the results could be used for evaluation of the treatment responses in both animal models and clinical cases (**Figure 5B**).

Until now, there have been no record of clinical trials (clinicaltrails.gov; www.clinicaltrialsregister.eu) and only three clinical studies have been reported regarding the effect of small molecules on IVD regeneration: Five human patients with non-small cell lung cancer and IDD received gefitinib treatment over the past five years, which not only resulted in tumor regression but also ameliorated IDD 20. Makris *et al*. conducted a retrospective cohort study to evaluate the use of statins in higher doses for patients suffering from a spinal degenerative joint disease (SDJD). They described an inversely proportional relationship between the dose of statins prescribed to patients suffering from hypercholesterolemia and the risk of SDJD occurrence 119. Contrary to the previous reports, a higher risk of developing low back pain was associated with the higher dosage of statins, which tended to cause statin-induced myopathy 120. With respect to the *in vivo* and clinical studies, the application of biological treatment strategies for the regeneration of IVDs remains a largely undiscovered field which has great potential for further investigations.

## Future prospective and Conclusions

Small molecule drugs have shown promising therapeutic potential for the regeneration of IVD tissue and attenuation of the degenerative process. Nowadays, the focus in medicine is on individualized therapies and the factors aggravating the degenerative process in an individual patient need to be considered in more detail. Research towards therapies that induce the intrinsic regenerative potential of the IVD and CEP cells are of high interest due to the long-lasting effects and limited disturbance of the tissue homeostasis which happens with external manipulation. Several *in vitro* studies have investigated the potential of small molecules to induce autophagy, for stimulating the natural process of IVD cells to adapt and regenerate in a stressful environment 83, 88. To effectively control the IDD process, emphasis should be put upon an individualized therapeutic approach, like estradiol supplementation which has been advised for menopausal women 121. Studies on senomorphic drugs show the promising potential of curcumin or o-vanillin in reducing the number of senescent cells. A selective apoptotic effect on senescent cells, while sparing the actively proliferating cells is of high significance and further work must be followed up to translate this promising potential to organ culture or *in vivo* studies 104. An important factor which should be highlighted for utilization of different small molecule agents is the limit point of pharmacological or biological treatments. The selection of degeneration severity-based therapeutic options needs to be assessed before the start of any treatment. Biological or molecular treatments including growth factors, cell, gene therapy, and small molecules, could typically be used to repair and regenerate the degenerated disc in the early stages of IDD (Pfirrmann grade I-III). However, these methods are not sufficient to treat the advanced stages of diseases (Pfirrmann grade IV-V) and surgery would be required as the last resort. Therefore, detailed diagnostic measures are indispensable to assess the type and stage of IDD. New diagnostic approaches (i.e., *in vivo* bioluminescence) should be developed to detect early IDD or to evaluate the prompt tissue responses to different small molecules *in vivo*.

The promising results of the discussed investigations are only the foundation to answer the more complex questions arising from *ex vivo* and *in vivo* models. Small molecules have a huge therapeutic potential, as their application can be directed with controlled release formulas and in many cases, the evidence of their efficacy is already proven in the treatment of other diseases in the clinics 61, 92, 119. It is of great importance to consider the dosing, the systemic effects of the drugs and interactions of the various compounds with each other and with over the counter and prescribed medicines. Combination therapies may further potentiate the positive effects of the small molecules on the regeneration of the IVD cells. In the search for therapies on the degenerated IVD, delivery of the compound to the IVD tissue is a question that will always complicate the path to clinical translation (Table S4). The small molecules administered systemically may be able to reach the disc in some cases; however, in compromised situations such as CEP damage or sclerosis, it may be a challenge to reach an effective concentration of the compound in the IVD. Local delivery of small molecules (i.e., direct injection) is another strategy to overcome the limitations of systemic delivery, such as diffusion problem due to CEP damage, or systemic side effects. The evolving field of hydrogels, microspheres, nanoparticles, and further substances that allow for a controlled release *in situ* show promising results to resolve this problem in the future 20, 61, 114. Nevertheless, needle size should be considered to avoid exacerbating degeneration.

*In vitro* results have shown the beneficial effects of small molecules on regeneration of degenerated IVDs through attenuation of inflammation, oxidative stress, apoptosis, catabolism, and stimulation of anabolic processes via different signaling pathways. Similarly, most *in vivo* results also indicate beneficial effects of these small molecules on the amelioration of IDD 20, 57, 63, 92, 116. However, the number of clinical studies in this regard is very low, and no clinical trials on the effect of these small molecules on IDD have been performed so far. In future, a road map for discovery of new small molecules and their clinical translation for IDD treatments should be provided. To date, the major limitations for clinical translation include the insufficient *in vivo* evidence, due to a lack of representative animal models; the lack of an adequate delivery system for different small molecules; and the lack of patient stratification methods to identify the patients who would respond to and benefit from a small molecule-based therapy. The potential necessity of long-term treatment should also be emphasized to achieve a successful therapy from small molecule agents for regeneration of degenerated IVD. The avascular nature of the IVD may increase the duration of treatment, particularly for oral administration of small molecules which should first be absorbed by digestive tracts, delivered to the target site via the systemic circulation and finally reach the degenerated disc through diffusion.

To bridge the gap between rudimentary animal models and clinical studies, a more clinically relevant animal model should be used to better replicate the complexity of the human IVD and the pathology of IDD. The modeling of pain or inflammation, systemic response to degeneration and related treatment are only represented to a limited extent in the established *in vitro* models. Considerations for establishing an appropriate animal model include factors like the absence of notochordal cells, size of IVD tissue, body mass relative to humans, mechanical compression forces upon the IVD, type of injury and ethics 113. Non-human primates (i.e., baboons and macaques) and chondrodystrophic dogs (i.e., beagle and dachshund) closely match the clinical condition of IDD regarding many of the physical and mechanistic criteria (spontaneous models of IDD) 122, 123. However, ethical considerations should preclude their widespread use in pre-clinical studies. The ovine IDD model could be one of the best animal models due to desirable characteristics such as the absence of notochordal cells, similar body mass to humans, and mechanical compression forces acting upon the IVD 124. Other study parameters such as follow-up time and animal numbers are also important factors in study design. With short-term follow-up (8-12 weeks), the study is usually limited to investigating the protective effect of small molecules on IDD progression; while longer-term follow-up studies (6 to 12 months) are necessary to detect any regenerative effects 97. Moreover, if ethical considerations allow for it, a higher number of animals for *in vivo* studies could be used to decrease the outcome variation and make it easier to draw conclusions 125.

Behavioral pain assessment should be utilized to assess the effect of small molecules on IDD such as von Frey filament test, gait analysis, weight loading, and hot plate analysis. It should be highlighted that the collaboration of neuroscience, orthopedic, and biomedical sciences is a necessity to extend the current understanding of the complex and multifactorial pathophysiology of IDD and discogenic pain 110. For *in vivo* imaging techniques, using equipment with higher sensitivity and resolution is also recommended. Recently, better MRI systems have become available for small animal models that are able to generate magnetic fields of 9.5T or even 21T, offering an excellent tool for small animal preclinical studies 111. Regarding ethical issues, it should also be noted that all mechanisms, pathways and related beneficial effects of each small molecule on IDD have to be identified as best as possible by *in vitro* studies, followed by ex-vivo IDD organ culture models to confirm the results. Furthermore, new experimental techniques such as lab-on-a-chip that simulate the activities, mechanics and physiological response of entire organs and organ systems are being developed 126**.** Recently, a microfluidic disc-on-a-chip device has been developed that was tailored for laboratory small animal disc organs as a long-term ex-vivo organ culture platform. This device lays groundwork for future studies by simulating the chronic nature of IDD 126.

In conclusion, small molecule therapy is an alternative treatment for conventional therapies and surgical approaches in discogenic pain. Effective regeneration of the degenerated IVD with small molecules has opened an important area of research in IVD regeneration in the last decade. However, it is of great importance to investigate the mechanism of action of small molecules in targeting different signaling pathways towards an effective therapy. Several of these small molecules are effective in inhibiting apoptosis, inflammation, oxidative stress, and senescence, which can prevent degeneration of the disc cells. They also showed anabolic and anti-catabolic effects (intrinsic regeneration) which are critical factors for the regeneration of damaged IVD. Further analysis, especially in large animal and advanced organ culture models and then clinical studies are needed to confirm the preliminary results obtained from *in vitro* investigations. Moreover, it will be of importance to investigate other regenerative effects of different small molecules, such as the induction of NP-like cell differentiation in endogenous and exogenous stem or progenitor cells.

## Supplementary Material

Supplementary figures and tables.

## Figures and Tables

**Table 1:** Mechanism of action of small molecules used for IVD regeneration

Small molecule	Anti-apoptotic	Anti-inflammatory	Anti-oxidative	Anti-catabolic	Anabolic	Miscellaneous
**Natural origin**						
Cannabidiol	58	58, 127	58			
Epigallocatechin 3-gallate	50	50, 78	50, 78	50		
Naringin	63, 128	87, 128	63, 128	63, 87, 129	63, 87, 129	
Urolithin A		54	130			
Rhein	67	55, 67	67	67		
Estradiol	89, 131	65, 132, 133			132, 134	
Curcumin		135		135		104 Senolytic; 102 mTOR inhibitor.
o-Vanillin		104				104 Senolytic
Icariin	80, 136-136	136	80, 136, 137	138	80	
Resveratrol	82, 139	82, 91, 140	82, 91, 139	82, 140	81, 82, 91	
Celecoxib		61, 141		61		
Kaempferol	52	52			52	105 BMP2 activator
Berberine	70, 71, 86	70	86	70, 71, 86		
Luteoloside	57	57	57	57	57	
**Chemical/ Synthetic**						
Statins		142			107, 142	106 BMP2 activator
Metformin	92	59		92	92	92 Autophagy
APO866	93	93				93 Autophagy
Dexmedetomidine						74 Inhibit pyroptosis
SM04690		109			113	[109]Wnt pathway Inhibitor
Gefitinib		20			20	20 Autophagy
Tofacitinib		56			56	
INK-128						102 mTOR inhibitor
NVP-BEZ235						102 mTOR inhibitor
MK-2206						102 mTOR inhibitor

**Table 2:** Study setup of different investigations using small molecules for IVD regeneration

Small molecule	*In vitro* (Cell culture)	*Ex vivo* (Organ culture)	*In vivo*	Clinical study
**Natural origin**				
Cannabidiol	58		Rat 127	
Epigallocatechin 3-gallate	50, 78		Rat 50	
Naringin	63, 87, 128, 129		Rat 63	
Urolithin A	143		Rat 54	
Estradiol	89, 90		Rat 121, 132	
Curcumin	144		Rat 145	
o-Vanillin	104			
Icariin	80, 136-138		Rat 146	
Resveratrol	82, 139, 140	91	Rabbit, Rat 91, 117	
Celecoxib	61, 141		Dog 61, 115, 141	
Kaempferol	52			
Berberine	70, 71		Rat 71	
Luteoloside	57		Rat 57	
**Chemical/Synthetic**				
Statins	107		Rat 142	118, 119
Metformin	92		Rat 92	
APO866	93			
Dexmedetomidine	74			
SM04690	109		Rat 109	
Gefitinib	20		Rat 20	20
Tofacitinib	56, 60	56		
INK-128	102			
NVP-BEZ235	102			
MK-2206	102			

**Table 3:** An overview of the *in vivo* studies for individual small molecules

Molecule	
**Naringin**	
***Study***	*** Zhang et al. 2018 63***
Aim	To assess the potential therapeutic effect of Naringin on IVD regeneration
Animal/Patient-model	Sprague-Dawley Rat (G: NI, n=36), puncture-induced rat IDD model
Intervention	Intraperitoneal injection of Naringin (80mg/kg/day)
Analysis	Histopathology and MRI at 4 and 12 weeks after surgery
Results/Conclusion	**Histology:** protection of CEP, no significant difference between saline (vehicle) and Naringin treated groups regarding the damage to NP cells after 12 weeks; **MRI:** There is a significant difference between (vehicle) and Naringin treated groups in terms of Pfirrmann MRI grade scores. *Conclusion:* Naringin may exert a protective effect on IVD after an initial injury.
**Cannabidiol (CBD)**	
***Study***	*** Silveria et al. 2014 127***
Aim	To assess the protective effect of CBD on lesion-induced IDD
Animal/Patient-model	Wistar Rat (G: male, n=19), puncture-induced rat IDD model
Intervention	Intradiscal injection of CBD (60-80-120nm)
Analysis	Histopathology (15 days after surgery), MRI (2 and 15 days after surgery)
Results/Conclusion	**Histology:** CBD (120 nmol) prevented the typical histological changes in the AF, no significant protective effect seen on NP. **MRI:** Injection of CBD (120 nmol) immediately after lesion significantly improved MRI pixel intensity. *Conclusion:* Considering that CBD presents an extremely safe profile, only high dose of CBD (120 nmol) could halt the IDD progression.
**Epigallocatechin 3-gallate (EGCG)**	
***Study***	***Krupkova et al. 2014 50***
Aim	To analyze the effect of EGCG on discogenic pain
Animal/Patient-model	Sprague-Dawley Rat (G: female, n=60), Autologous NP was harvested from the tail and applied to the dorsal root ganglion (DRG, L5-L6)
Intervention	Local injection of 0.1ml EGCG (10 and 100μM) into the underlayer of epineurium
Analysis	Hind paw withdrawal response to von Frey Filament test (2, 7, 14, 21 and 28 d post-surgery)
Results/Conclusion	**von Frey Filament test:** During 28 days, NP+EGCG treatment significantly increased mechanical withdrawal thresholds in comparison to the NP+vehicle group, and reached levels measured in the sham group. *Conclusion:* EGCG (10 and 100μM) inhibits pain behaviour *in vivo.*
**Urolithin A (UA)**	
***Study***	***Liu et al. 2018 54***
Aim	To assess the beneficial effect of UA on IDD
Animal/Patient-model	Sprague-Dawley Rat (G: male, n=30), puncture-induced rat IDD model
Intervention	Oral delivery of UA (0.25 g per kg of diet or 25 mg/kg/day)
Analysis	X-ray, MRI and histopathology (4 weeks post-surgery)
Results/Conclusion	**X-ray:** UA treatment group showed no significant disc space. **MRI:** Pfirrmann grade scores were lower in the UA treatment group than the IDD control **Histopathology:** UA treatment group considerably alleviated IVD destruction in comparison to the IDD control *Conclusion:* UA may be a useful small molecule for the treatment of IDD.
**Estradiol (E2)**	
***Study***	*** Jin et al. 2018 121***
Aim	To analyze the effect of E2 on IDD in the model of menopause rats
Animal/Patient-model	Sprague-Dawley Rat (G: female, n=30), oophorectomy (OVX) to induce menopausal in rats
Intervention	10 µg/kg/day E2 supplementation for 12 weeks
Analysis	MRI, histopathology, IHC (LC3 for autophagy) (12 weeks post-surgery)
Results/Conclusion	**MRI:** T2 mapping showed a marked increase in results in OVX + E2 and sham when compared to OVX + vehicle **Histopathology:** The OVX + E2 treatment group showed the NP tissues were similar to those observed in the sham group **IHC:** There are no significant differences between OVX + E2 treatment and sham group in terms of autophagy *Conclusion:* E2 via regulating the redox balance (autophagy) of IVD could be a potential therapeutic agent for IDD in the postmenopausal women.
***Study***	***Liu et al. 2018 132***
Aim	To further explore whether estradiol (E2) had protective effects on IDD in OVX rats
Animal/Patient-model	Sprague-Dawley Rat (G: male, n=40), puncture-induced OVX-rat IDD model
Intervention	Subcutaneous injection of 20 µg/kg/day E2 for 28 d
Analysis	X-ray (disc height index-DHI), histopathology, IHC, western blot (WB) (30 d post-surgery)
Results/Conclusion	**X-ray:** In OVX + E2 treated animals, X-ray showed a markedly higher DHI in comparison to the OVX+ vehicle group. **Histopathology:** Mean histological scores in Sham and OVX + E2 group were significantly lower than OVX+ vehicle group **IHC:** E2 downregulated caspase-3, MMP-3 and MMP-13 proteins level but upregulated collagen Type II **WB:** Confirmed IHC results *Conclusion:* E2 shows protective effects against IDD by down-regulating catabolic proteins and up-regulating anabolic ones in OVX- animal models.
**Icariin**	
***Study***	***Hua et al. 2020 146***
Aim	To explore the effect of icariin on IDD
Animal/Patient-model	Sprague-Dawley Rat (G: male, n=24), needle puncture model
Intervention	Intraperitoneal administration of icariin (30 mg/kg) for 8 w post-surgery
Analysis	MRI and histopathology (8 weeks post-surgery)
Results/Conclusion	**MRI:** Pfirrmann grade scores were significantly lower in the icariin treatment group than the saline treatment **Histopathology:** Icariin treatment reduced histopathological changes (disruption of AF), although some degeneration was still observed *Conclusion:* Icariin could be utilized as a protective agent to inhibit further degeneration after injury.
**Resveratrol**	
***Study***	***Kwon 2013 91***
Aim	To evaluate whether resveratrol had anabolic effects on IDD in a rabbit model
Animal/Patient-model	New Zealand white rabbit (G: male, n=24), needle puncture model
Intervention	Two times intradiscal injections of 15 µL of 100 µM resveratrol in DMSO, repeat dose administrated 2 weeks after the first injection
Analysis	MRI (4, 8, 16 weeks after the initial injection), histopathology (16 weeks after the initial injection)
Results/Conclusion	**MRI:** MRI scores significantly lower in the resveratrol group than the DMSO (vehicle) group **Histopathology:** Significant higher histological grades are noted in the DMSO group when compared with the resveratrol group *Conclusion:* icariin may be a promising candidate for the treatment of IDD.
***Study***	***Lin et al. 2016 117***
Aim	To assess the effect of resveratrol of on NP-mediated (discogenic) pain
Animal/Patient-model	Sprague-Dawley Rat (G: female, n=36), NP-mediated radiculopathy (model)
Intervention	Local injection of 0.1ml resveratrol (50 µM) into the underlayer of epineurium
Analysis	von Frey filaments test (0, 3, 7, 14, 21 d post-surgery), histopathology and IHC (7 and 14 d post-surgery).
Results/Conclusion	**von Frey filaments test:** significant pain reduction by resveratrol treatment **Histopathology and IHC:** resveratrol treatment showed improved cell structure, with decreased edema and focal hyperemia compared with the negative control group. The expression level of IL-1 and TNF-α proteins decreased by resveratrol treatment. *Conclusion:* The results indicate the potential of resveratrol for attenuating discogenic pain.
**Celecoxib (CXB)**	
***Study***	***Willems et al. 2015 141***
Aim	To assess the effect of controlled delivery of CXB on IVD regeneration
Animal/Patient-model	Dog (G: female, n=18), canine model of spontaneous mild IDD
Intervention	a bolus intradiscal injection of CXB (7.7 μM), intradiscal injection CXB loaded hydrogel (77 μM and 770 μM)
Analysis	Histopathology and IHC, Q-PCR (4 weeks after the initial injection)
Results/Conclusion	**Histology and IHC:** No significant differences were found between the injected treatments **Q-PCR:** Only relative gene expression levels of BCL2 and PGE2 were significantly downregulated in the CXB-loaded hydrogel in comparison to the sham *Conclusion:* The controlled delivery of CXB resulted in limited inhibition of PGE2 production in dogs with spontaneous IDD *Limitations:* Due to technical limitations, it was impossible to determine the CXB tissue levels, and hence *in vivo* release profile of CXB.
***Study***	***Tellegen et al. 2018 61***
Aim	The effect of control release of CXB on IVD regeneration
Animal/Patient-model	Dog (G: male, n=6), canine IDD model
Intervention	One month after surgery, Intradiscal delivery of 40 µl CXB loaded microsphere (CXB-M), low (8.4 µg CXB) and high dose (280 µg CXB)
Analysis	MRI (0 d, 4 and 12 weeks after injection), histopathology and IHC (12 weeks after the initial injection)
Results/Conclusion	**MRI:** DHI was maintained in the disc treated with either low or high dose CXB-M, Pfirrmann score was lower in CXB-M treated groups compared to the negative control **Histopathology and IHC:** Controlled release of CXB inhibited progression of IDD, the development of osteophyte formation, and decreased the immunopositivity of nerve growth factor *Conclusion:* Intradiscal controlled release of CXB inhibited progression of IDD *in vivo.*
***Study***	***Tellegen et al. 2018 115***
Aim	To assess the impact of sustain delivery of CXB on discogenic pain
Animal/Patient-model	Dog (G: female, n=10), canine patients with low back pain
Intervention	Intradiscal injection loaded hydrogel containing 2.93 μg/mL CXB
Analysis	MRI (0 d and 12 weeks after injection), clinical examination of low back pain (12 weeks after the initial injection)
Results/Conclusion	**MRI:** No evident of CXB regenerative effects on MRI **Clinical examination:** The reduction of back pain achieved in 9 of 10 dogs. In 3 of 10 dogs, back pain recurred after 12 weeks *Conclusion:* the majority of the treated canine patients, quality of life improved without evident regenerative effects *Limitations:* small group size, absence of a placebo group.
**Berberine**	
***Study***	***Luo et al. 2019 86***
Aim	The effects of berberine on IDD were investigated
Animal/Patient-model	Sprague-Dawley Rat (G: female, n=24), needle puncture model
Intervention	Intraperitoneal administration of berberine (150 mg/kg/day) for 8 weeks post-surgery
Analysis	MRI and histopathology (8 weeks post-surgery)
Results/Conclusion	**MRI:** Pfirrmann scores were significantly lower in the berberine treated animals than the saline treatment **Histopathology:** The histological scores in the berberine treatment group significantly lower than IDD control group (saline). *Conclusion:* Berberine could attenuate puncture-induced IDD in animal model.
**Metformin**	
***Study***	***Chen et al. 2016 92***
Aim	To assess the effects of Metformin on IDD
Animal/Patient-model	Rat (G: NI, n=NI), puncture-induced IDD model
Intervention	Intraperitoneal administration of metformin (50 mg/kg/day) for 16 weeks post-surgery
Analysis	MRI and histopathology (8-16 weeks post-surgery)
Results/Conclusion	**MRI:** Metformin treated group showed lower Pfirrmann scores compared to the vehicle-treated animals **Histopathology:** The histologic score of the metformin group was significantly lower than those of negative control both at 8- and 16-weeks post-surgery *Conclusion:* Metformin showed a protective effect against progression of IDD.
**SM04690**	
***Study***	***Barroga et al. 2017 109***
Aim	To investigate the effects of SM04690 on IDD
Animal/Patient-model	Rat (G: NI, n=NI), puncture-induced IDD model
Intervention	Single intradiscal of SM04690 (0.066 mg/disc)
Analysis	X-ray and histopathology (6 weeks post-surgery)
Results/Conclusion	**X-ray:** % DHI in SM04690 treated animals significantly increased compared to vehicle control **Histopathology:** Treatment by SM04690 increased number of NP cells and increased ECM vs. vehicle control *Conclusion:* SM04690 has potential as a modifying therapy for IDD.
**Gefitinib**	
***Study***	***Pan et al. 2018 20***
Aim	to investigate the therapeutic potential of gefitinib in ameliorating IDD
Animal/Patient-model	Sprague-Dawley Rat (G: female, n=18), puncture-induced IDD model
Intervention	Three µl aliquots intradiscal injection of gefitinib (30 mM)
Analysis	MRI and histopathology (4 weeks post-surgery)
Results/Conclusion	**MRI:** DHI% values of the gefitinib-treated group were significantly higher than those of the IDD control. The Pfirrmann scores also showed that the degree of disc degeneration was markedly lower in the gefitinib-treated group as well. **Histopathology:** The gefitinib treatment considerably decreased the histological scores in comparison to IDD control group. *Conclusion:* The results suggest the potential application of gefitinib for treating IDD.
**Statin**	
***Study***	***Than et al. 2014 142***
Aim	To find a new conservative treatment for IDD and related discogenic pain
Animal/Patient-model	Sprague-Dawley Rat (G: NI, n=272), puncture-induced IDD model
Intervention	Six weeks post-surgery, intradiscal injection of 2μL simvastatin (SIM) at 3 different doses (5, 10, or 15 mg/mL) in either a saline or hydrogel carrier
Analysis	MRI and histopathology and IHC (2, 4, 8, 12 and 24 weeks after the initial injection)
Results/Conclusion	**MRI:** MRI analysis showed a higher index (better results) for treatment with 5 mg/ml SIM administered in comparison to the higher doses (15 mg/ml), MRI index: 5 mg/ml hydrogel>5 mg/ml saline>10 mg/ml saline>15 mg/ml saline>15 mg/ml hydrogel **Histopathology and IHC:** histological grades confirmed the MRI results *Conclusion:* Intradiscal injection of simvastatin into IDD may result in retardation of degeneration process (5 mg/ml simvastatin in a hydrogel carrier) *Limitation:* unbalanced time point analysis for all groups, Control group was assessed only histologically.
**Luteoloside**	
***Study***	***Lin et al. 2019 57***
Aim	To investigate the protective potential of luteoloside in IDD
Animal/Patient-model	Sprague-Dawley Rat (G: NI, n=36), puncture-induced IDD model
Intervention	Intraperitoneal injection of 10mg/kg/day luteoloside for 4 and 8 weeks post-surgery
Analysis	MRI, X-ray and histopathology (4, and 8 weeks post-surgery)
Results/Conclusion	**MRI:** Pfirrmann MRI grade scores were significantly lower in the luteoloside group than in the IDD group **X-ray:** DHI was significantly lower in the IDD group than in the luteoloside treatment group **Histopathology:** Both ECM and NP tissues were better preserved in the luteoloside-treated group when compared to the IDD group *Conclusion:* Luteoloside only ameliorate IDD progression during long-term follow-up (8 weeks).
**Curcumin**	
***Study***	***Ma et al. 2015 145***
Aim	To observe the effect of curcumin on IDD
Animal/Patient-model	Sprague-Dawley Rat (G: male, n=60), Surgically induced IDD model in the lumbar area (removal of the spinous processes, the articular processes, the supraspinous ligaments and the interspinous ligaments).
Intervention	Intraperitoneal injection of 50mg/kg and 100mg/kg curcumin (single dose)
Analysis	MRI, Electron microscopy (EM), RT-PCR, and western blot (WB) (6 weeks post-surgery)
Results/Conclusion	**MRI:** The IVD signals of curcumin-treated animals (L1-6) were slightly lower than those in the normal group but were considerably higher than those of IDD models. **EM:** The degree of degeneration related to NP, AF and ECM structure of IVD samples was better in curcumin-treated animals in comparison to the IDD models **RT-PCR:** The expression levels of NF-κB-p65 and TNF-α were significantly lower in curcumin-treated animals than the other groups. **WB:** curcumin-treated animals had significantly lower NF-κB-p65 and TNF-α expression levels than IDD animal models. *Conclusion:* curcumin can decelerate the IDD process by blocking the NF-κB-p65 pathway and reducing inflammatory factors *Limitation:* 1. No statistical analysis was performed on the differences between each group regarding the MRI test. 2. Lack of further verification of the type of lumbar IDD and related IDD categorization.

G: gender, NI: no information.

## Abbreviations

LBP: low back pain
IVD: intervertebral disc
IDD: intervertebral disc degeneration
CLBP: chronic low back pain
NP: nucleus pulposus
AF: annulus fibrosus
SP: spinous process
TP: transverse process
SC: spinal cord
VB: vertebral body
ECM: extracellular matrix
PG: proteoglycan
IFN-γ: interferon-gamma
TNF-α: tumor necrosis factor-alpha
IL: interleukin
ADAMTS: a disintegrin and metalloproteinase with thrombospondin motifs
MMP: matrix metalloproteinase
CBD: cannabidiol
ECGC: epigallocatechin gallate
NF-κB: nuclear factor kappa-light-chain-enhancer of activated B cells
MAPK: MAP Kinase
JAK3: janus kinase 3
Nrf2: nuclear factor erythroid 2-related factor 2
HO-1: heme oxygenase-1
PGE2: prostaglandin E2
COX2: cyclooxygenase-2
E2: 17 beta-estradiol
Bcl-2: B-cell lymphoma 2
Bax: BCL-associated X
BAK1: BRI1-associated receptor kinase 1
PI3K: phosphatidylinositol 3-kinase
AKT: protein kinase B
ROS: reactive oxygen species
iNOS: inducible nitric oxide synthase
SOX: SRY-Box transcription factor
TGF-β: transforming growth factor beta
EGFR: epidermal growth factor receptor
BMP: bone morphogenetic protein
MRI: magnetic resonance imaging
RT-PCR: real-time polymerase chain reaction
IHC: immunohistochemistry
WB: western blot
HIVD: healthy intervertebral disc
DIVD: degenerated intervertebral disc
SMs: small molecules
CXB: celecoxib
PCLA-PEG-PCLA: poly(ε-caprolactone-co-lactide)-b-poly(ethylene glycol)-b-poly(ε-caprolactone-co-lactide)
mTOR: mammalian target of rapamycin
NLRP3: nod-like receptor protein 3
IGF-I: insulin-like growth factor 1
PDGF: platelet-derived growth factor
FGF: fibroblast growth factor
ERK: extracellular signal-regulated kinase
SDJD: spinal degenerative joint disease
UA: urolithin A
T: Tesla
DHI: disc height index
Ab: antibody
NE: neo-epitope
DC: disc cell
IP: intraperitoneal injection
ID: intradiscal injection/delivery
