# Supplementary material for: Small molecule-based treatment approaches for intervertebral disc degeneration: Current options and future directions
Source: Theranostics. 2021 Jan 1;11(1):27–47. doi: 10.7150/thno.48987 (PMC7681102; doi:10.7150/thno.48987)
Supplement: Supplementary file 1 — Supplementary figures and tables. [file thnov11p0027s1.pdf]

Table S1. The major advantages and potential limitations of small molecules in comparison to larger molecules

Advantages	Limitations
**High potency**<br>Small molecules can be developed to have a significant therapeutic impact at low dose when compared to larger molecules	**Easy to replicate in market**<br>Small molecules simplicity leaves them more open to competition from generic equivalents. On the other hand larger, more complex drugs are harder to replicate and therefore face less competition
**Cost-effective production**<br>Small molecules are effective at smaller amounts of their active pharmaceutical ingredient (API), therefore they are more cost effective in comparison to the large molecule drugs	**Low Specificity**<br>Basically, the specificity of small molecules is lower than larger molecules such as peptides or other biological agents
**High quality**<br>Today, the technology for developing several small molecules is advanced in pharmaceutical companies to maintain the exceptional reproducibility and the original efficacy	
**Oral delivery**<br>The small molecular weight and size of small molecules means they can be absorbed easily via digestive tracts. Consequently, they can be prescribed in oral form without the requirement of injection	
**Stability**<br>In contrast to several biological agents, most small molecules typically do not need the cold chain assurance required by large molecule drugs which improves their shelf-life and shipping process	

Table S2. An overview of small molecules used for IVD regeneration

Small molecule	*Molecular weight*	*Definition*
Natural origin	g/mol	
Cannabidiol	314.46	The major nonpsychotropic phytocannabinoid of the marijuana plant (Cannabis sativa). In contrast to major cannabinoids such as Δ9-THC, CBD does not cause any psychotomimetic or cognitive effects
Epigallocatechin 3-gallate	458.37	A biologically active polyphenolic catechin found in green tea (*Camellia sinensis*)
Naringin	580.54	A natural flavonoid found in citrus fruits particularly grapefruit where Nar is responsible for the fruit's bitter taste
Urolithin A	228.20	Urolithins, which are thought to be the intestinal microbial metabolites of both ellagitannins and ellagic acid, include urolithin A, urolithin B, urolithin C and urolithin D.
Rhein	284.22	Also known as cassic acid, is a substance in the anthraquinone group obtained from rhubarb. Like all such substances, rhein is a cathartic.
Estradiol	272.36	An estrogen steroid hormone and the major female sex hormone
Curcumin	368.38	For centuries the root curcuma longa L. (diferuloylmethane) has been used in traditional Chinese and Indian medicine for the treatment of diabetic wounds, hepatic disorders rheumatism and sinusitis
o-Vanillin	152.15	The metabolites of curcumin with better bioavailability, water solubility and chemical instability which therefore gained importance in research
Icariin	676.66	The natural flavonoid glucoside which has been used in traditional Chinese Medicine as the herb *Epimedium brevicornum* in the treatment of a variety of diseases
Resveratrol	228.24	The potent antioxidant and natural polyphenol which is produced by numerous plants as a phytoalexin, in defense of the plant against irradiation and infectious agents, and is found, as a result in many fruits such as grapes skin and seeds, and therefore whine, as well as peanuts in high amounts
Celecoxib	381.37	Cyclooxygenase Inhibitors have been successfully in use for the treatment of pain and inflammatory disorders, including the degenerative disc disease, since many decades
Kaempferol	286.23	A flavonoid agent which has been used as an herb in traditional Asian medicine for centuries for the treatment of abdominal pain, hypertension and headaches, as well as rheumatism.
Berberine	336.36	The isoquinoline alkaloid which is a plant extract that exerts anti-inflammatory, anti-oxidative and anti-apoptotic properties and is used as an herbal medicine in a variety of diseases
Chemical/ Synthetic		
Statins	404.54-558.64	These drugs were initially developed to treat hyperlipidaemia, they had more pleiotropic effects from cardiovascular to bone regeneration which are mediated by inhibition of 3-hydroxy-3-methylglutaryl coenzyme A (HMG-CoA) reductase
Metformin	129.16	Developed in 1922, Met has been the widely used oral medication for type II diabetes worldwide
APO866	391.51	Also known as FK866, is an inhibitor of nicotinamide phosphoribosyltransferase
Dexmedetomidine	200.28	An anxiety reducing, sedative, and pain medication
SM04690	505.55	A small-molecule inhibitor of the Wnt pathway
Gefitinib	446.90	A Food and Drug Administration-approved small molecule which inhibits epidermal growth factor receptor (EGFR) activity by competing with ATP binding to the receptor's kinase pocket
Tofacitinib	312.36	(Or CP-690,550) an orally administered JAK antagonist that is in development for the treatment of rheumatic arthritis and other immune disorders
Luteoloside	448.37	(Or Cynaroside) a flavone, a flavonoid-like chemical compound
INK-128	309.33	(Or Sapanisertib) a potent and selective mTOR inhibitor
NVP-BEZ235	469.54	(Or Dactolisiban) an imidazoquinoline derivative acting as a PI3K inhibitor
MK-2206	407.47	An allosteric AKT inhibitor which used for treatment of cancer

Table S3. Effective concentrations of small molecules *in vitro* for the regeneration of disc cells

**Small molecule**	Effective concentration (*in vitro*)	Ref.
Natural origin		
Cannabidiol	5 µM	[58]
Epigallocatechin 3-gallate	10 µM<br>10 µM	[50]<br>[78]
Naringin	20 µg/ml<br>60 µM<br>20 µg/ml<br>100 µM	[87]<br>[128]<br>[129]<br>[130]
Urolithin A	20 µM	[94]
Estradiol	1 µM<br>1 µM<br>1 µM<br>10 µM	[65]<br>[89]<br>[131]<br>[133]
Curcumin	20µM/ml	[145]
o-Vanillin	100µM	[104]
Icariin	40 µM	[138]
Resveratrol	200µmol/L<br>100 µM	[82]<br>[81, 140]
Kaempferol	100 µM	[52]
Berberine	25 µM	[53]
Chemical/ Synthetic		
Statins	3 µM (simvastatin)<br>5 µM (lovastatin)	[106]<br>[107]
Metformin	10 mM<br>200µM	[59]<br>[92]
APO866	10 nM	[93]
Dexmedetomidine	5 µM	[74]
SM04690	11 nM	[109]
Tofacitinib	2.5 mg/mL	[60]
Gefitinib	10 µM	[20]
Luteoloside	10 µM	[57]
INK-128	50 µM	[102]
NVP-BEZ235	50 µM	[102]
MK-2206	50 µM	[102]

Table S4. Potential clinical application of small molecules in IDD based on *in vivo* studies

**Potential clinical application**	Types of application	+/- Supplementation	Ref.
**Degenerated disc (grade I-III)**			
Cannabidiol	Local delivery (ID)	-	[127]
Naringin	Systemic delivery (IP)	-	[63]
Luteoloside	Systemic delivery (IP)	-	[57]
Urolithin A	Systemic delivery (PO)	-	[54]
Estradiol	Systemic delivery (PO, SC)	-	[121,132]
Curcumin	Systemic delivery (IP)	-	[145]
Metformin	Systemic delivery (IP)	-	[92]
Icariin	Systemic delivery (IP)	-	[146]
Resveratrol	Local delivery (ID)	-	[91]
Celecoxib (CXB)	Local delivery (ID)	CXB loaded microsphere	[61,141]
Berberine	Systemic delivery (IP)	-	[86]
Gefitinib	Local delivery (ID)	-	[20]
**Radicular and discogenic pain**			
Epigallocatechin 3-gallate	Local delivery (injection into underlayer of epineurium)	-	[50]
Celecoxib (CXB)	Local delivery (ID)	CXB loaded in hydrogel	[115]
SM04690	Local delivery (ID)	-	[109]
Statins	Local delivery (ID)	Statin loaded in hydrogel	[142]
Resveratrol	Local delivery (injection into underlayer of epineurium)	-	[117]

ID: Intradiscal, IP: Intraperitoneal, SC: Subcutaneous, PO: Peroral
